# Towards Better Understanding of KSHV Life Cycle: from Transcription and Posttranscriptional Regulations to Pathogenesis

**DOI:** 10.1007/s12250-019-00114-3

**Published:** 2019-04-25

**Authors:** Lijun Yan, Vladimir Majerciak, Zhi-Ming Zheng, Ke Lan

**Affiliations:** 1 State Key Laboratory of Virology, College of Life Sciences, Wuhan University, Wuhan, 430072 China; 2 National Cancer Institute, National Institutes of Health, Frederick, MD 21702 USA

**Keywords:** Kaposi’s sarcoma-associated herpesvirus (KSHV), Human herpesvirus 8 (HHV-8), Transcription, Posttranscriptonal regulation, ORF57

## Abstract

Kaposi’s sarcoma-associated herpesvirus (KSHV), also known as human herpesvirus-8 (HHV-8), is etiologically linked to the development of Kaposi’s sarcoma, primary effusion lymphoma, and multicentric Castleman’s disease. These malignancies often occur in immunosuppressed individuals, making KSHV infection-associated diseases an increasing global health concern with persistence of the AIDS epidemic. KSHV exhibits biphasic life cycles between latent and lytic infection and extensive transcriptional and posttranscriptional regulation of gene expression. As a member of the herpesvirus family, KSHV has evolved many strategies to evade the host immune response, which help the virus establish a successful lifelong infection. In this review, we summarize the current research status on the biology of latent and lytic viral infection, the regulation of viral life cycles and the related pathogenesis.

## Introduction

Kaposi’s sarcoma-associated herpesvirus (KSHV), also known as human herpesvirus-8 (HHV-8), is the etiological agent of Kaposi’s sarcoma (KS) and is also linked to two other lymphoproliferative disorders, primary effusion lymphoma (PEL) and a plasmablastic form of multicentric Castleman’s disease (MCD) (Chang *et al.*^1994^; Dupin *et al.*^1999^; Soulier *et al.*^1995^). Recent studies have reported a disease called KSHV inflammatory cytokine syndrome, or KICS, characterized by higher levels of interleukin-6 and high viral loads in patients (Uldrick *et al.*^2010^).

**Fig. 1:**
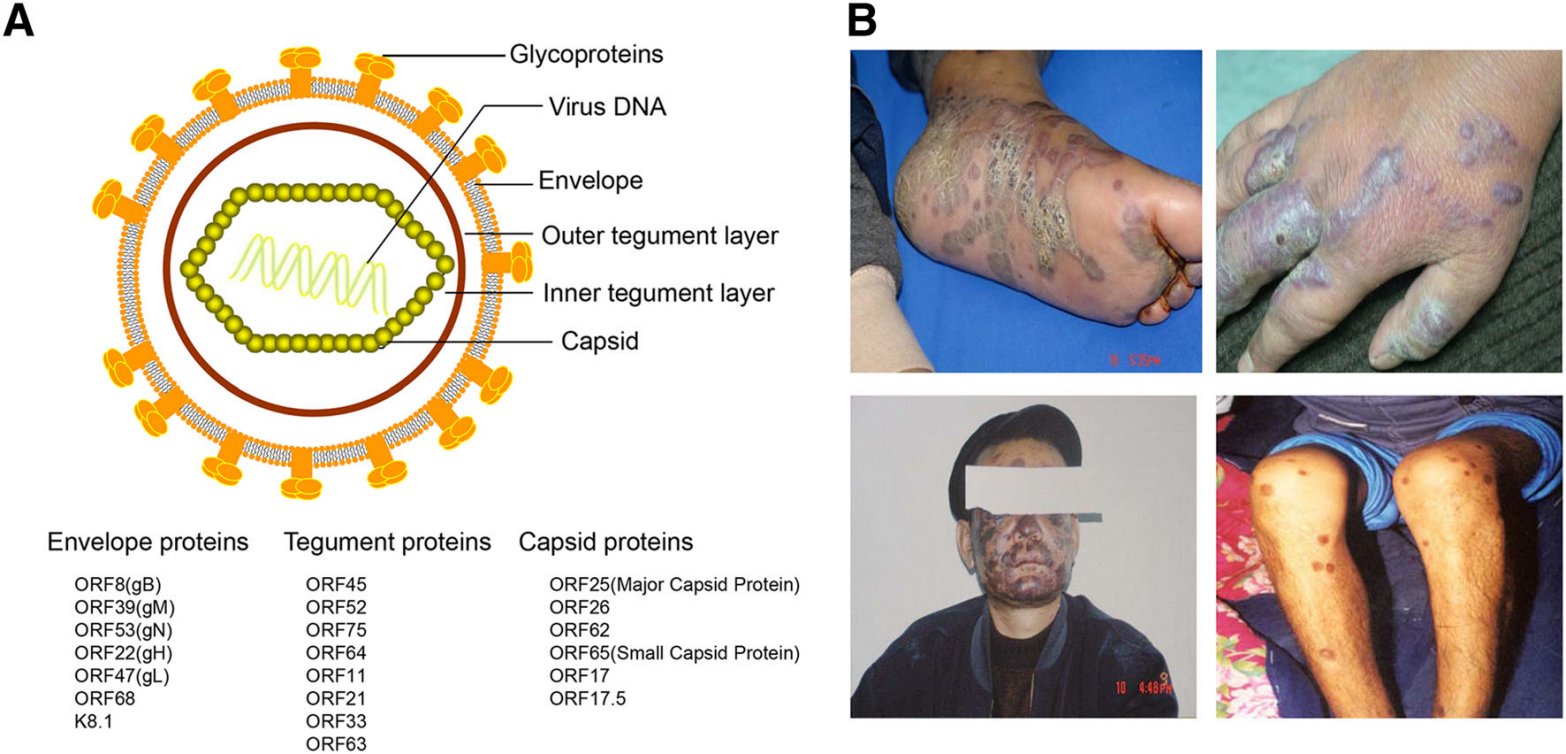
KSHV virion structure and clinical manifestations of Kaposi sarcoma. A Schematic presentation of KSHV structure. KSHV virions display the icosahedral nucleocapsids surrounded by a lipid bilayer envelope. Between the capsid and envelope is a morphologically amorphous layer called the tegument. The viral proteins found in KSHV capsid, tegument, and envelope are listed below. **B** Lesions of classical Kaposi’s sarcoma patients in Xinjiang province of China. The photos were provided by Dr. Tiejun Zhang from Fudan University School of Public Health.

KSHV is the γ2-herpesvirus, as it has a similar double-stranded linear DNA genome, but it varies in length from approximately 165–170 kb. The genome comprises a unique central coding region of approximately 145 kb and both sides of the region comprise highly GC-rich terminal repeats (TRs) (Russo *et al.*^1996^). KSHV has some highly conserved genes from open reading frame (ORF) 4 to ORF75 in a consecutive order. Similar to other herpesvirus members, these genes typically encode proteins associated with viral replication and structural virion components (Neipel *et al.*^1997^; Russo *et al.*^1996^). In addition, KSHV encodes a unique set of genes, designated with the prefix K (K1–K15), that have multiple functions in viral infection and virus-induced diseases (Ganem 1997; Russo *et al.*^1996^), a set of viral microRNAs (miRNAs), and several long non-coding RNAs including highly abundant lytic 1.1 kb polyadenylated nuclear RNA (PAN) (Staskus *et al.*^1997^). The basic structure of the KSHV virion is an electron-dense nucleocapsid surrounded by a lipid bilayer envelope. The layer between the envelope and capsid is called the tegument, which is divided into an outer and inner layer, although this division is not very clear. The envelope consists of seven glycoproteins, including ORF8 (glycoprotein B, gB), K8.1, ORF22 (gH), ORF47 (gL), ORF39 (gM), ORF53 (gN) and ORF68 (Akula *et al.*^2001^; Baghian *et al.*^2000^; Koyano *et al.*^2003^; Naranatt *et al.*^2002^; Wang *et al.*^2001^). The tegument proteins include ORFs 45, 52, 11, 21, 33, 63, 64 and 75, and are thought to possibly contribute to early viral replication events and genome entry upon primary infection (Bechtel *et al.*^2005^; Full *et al.*^2014^; Gillen *et al.*^2015^; Gregory *et al.*^2011^; Lan *et al.*2005b; Li *et al.*2016a; Lock *et al.*^2002^; Sathish *et al.*^2012^; Zhu *et al.*^2005^). In addition, several abundant host cytoplasmic proteins, such as HSP90, HSC70, EF-2B and actin are also found in the virions (Bechtel *et al.*^2005^) (Fig. 1A).

## Epidemiology of KSHV Infection

KS was first described as skin lesions, typically occurring among Mediterranean or Ashkenazi Jewish elderly men, by a Hungarian doctor named Moritz Kaposi in the late 19th century. Prior to the HIV epidemic, KS was found to be common only in certain geographical areas, such as the Mediterranean region and sub-Saharan Africa (Cook-Mozaffari *et al.*^1998^; Dollard *et al.*^2010^). However, with case reports of unusual KS occurring in young men having sex with other men (MSM) in the United States in the 1980s, KS became to be an AIDS-defining malignancy. In contrast to the speculation that HIV was the etiological agent of KS, Chang *et al.* first identified KSHV genomic DNA by performing representational differential analysis of KS lesions and normal tissue in 1994 (Chang *et al.*^1994^). To date, KSHV has been confirmed as the causative agent of KS.

According to reported clinical studies, KS is divided into four classes: classic KS, which develops especially in Jewish elderly men (Iscovich *et al.*^1998^); endemic KS, occurring in sub-Saharan Africa evenly in adults and children (Revilla-López *et al.*^2015^); AIDS-associated KS, closely associated with HIV infection; and iatrogenic KS, generally occurring in the recipients who receive the immunosuppressive therapy after organ transplantation.

Compared to other human herpesviruses, KSHV infection is not ubiquitous, and the incidence of KS is geographically limited from sub-Saharan Africa to Europe and the US. Numerous methods have been used to clearly illustrate data regarding the KSHV seroprevalence in large population-based studies (Gao *et al.*^1996^; Lennette *et al.*^1996^; Simpson *et al.*^1996^). Currently, KSHV seroprevalence varies among different populations and three major patterns are observable: (1) high-level endemic areas, mainly occurring in Africa with seropositivity rates greater than 50%; (2) intermediate-level endemic areas, mainly located in Mediterranean countries with seroprevalence rates between 10% and 25%; and (3) nonendemic areas, mainly referring to most parts of Europe, Asia and the US with no more than 10% seropositivity in the population. Additionally, the prevalence increases in gay men in Western countries and Amerindians are reported to be more susceptible to KSHV infection than other ethnic groups. In China, KSHV seropositivity varies among different regions and ethnicities, as the virus mainly infects individuals of Xinjiang province located in the northwest of China and adults of Kazak and Uyghur ethnicities, at seropositive rates ranging from 20% to 40%. Therefore, Xinjiang is considered an endemic KSHV region in China. Interestingly, KSHV has a higher seroprevalence in people of Han ethnicity in Xinjiang than in other Han populations in the rest of China, but the reason underlying the difference in the geographical and population-based distribution of KSHV incidence remains unknown (Cao *et al.*^2014^; Fu *et al.*^2009^; Liu *et al.*^2017^; Minhas and Wood 2014; Wang *et al.*^2010^; Zheng *et al.*^2017^) (Fig. 1B).

The modes of KSHV transmission vary among different regions and populations. In nonendemic regions, sexual contact is the main route of transmission, with evidence of an elevated prevalence in gay men (Martin *et al.*^1998^; Melbye *et al.*^1998^). However, there are no direct evidences that sexual contact among heterosexual individuals could possibly get the infection. Different studies are inconsistent about the transmission mode. Studies conducted among sexual workers in Africa have suggested that sexual transmission indeed occur in adults (Eltom *et al.*^2002^; Lavreys *et al.*^2003^), but another study conducted in South Africa showed no direct evidence of sexual transmission (Malope *et al.*^2008^). In addition, a study conducted among the Iranian general population and intravenous drug users (IVDUs) with high-risk sexual behaviors demonstrated that KSHV infection was more prevalent in the IVDUs compared with healthy subjects and the findings indicate the likelihood of sexual route of KSHV transmission in the Iranian population (Kakavand-Ghalehnoei *et al.*^2016^). Another study from China reported that KSHV prevalence is similar in HIV-negative heterosexuals, female sex-workers, and sexually transmitted infection (STI) male patients, but represents approximately 20% and 30% in IVDUs and MSM, respectively (Zhang *et al.*^2017^). The study also indicates that sexual transmission of the virus is important in MSM but not in heterosexuals (Zhang *et al.*^2017^).

Besides sexual transmission, peripheral blood analysis results suggest that KSHV can be transmitted via blood and blood products (Dollard *et al.*^2005^; Whitby *et al.*^1995^), although the viremia in KSHV infected patients was uncommon and the specific cell-associated nature didn’t support efficient transmission. However, studies conducted in Uganda, where HHV-8 is endemic, provided a strong evidence that KSHV is transmitted by blood transfusion (Hladik *et al.*^2006^). Further studies showed that transfusion of short stored blood presents increased risk of death (Hladik *et al.*^2012^). The high prevalence in children especially in endemic regions leads to the possibility of transmission from mother to child vertically and some reports support the hypothesis in part, but more samples and studies are needed to further exploration (Lisco *et al.*^2006^; Mantina *et al.*^2001^).

Apart from the routes discussed above, saliva exchange in a nonsexual manner may be an important mechanism of viral spread among children and adults in the endemic regions, as viral DNA is detectable in their saliva (Boldogh *et al.*^1996^; Martin *et al.*^1998^; Smith *et al.*^1999^).

## Life Cycles of KSHV

KSHV infects multiple cell types including B lymphocytes, endothelial and epithelial cells, typically via membrane fusion mediated by several viral glycoproteins, such as gB, gH and gL (Avitabile *et al.*^2009^; Krishnan *et al.*^2005^; Pertel 2002; Veettil *et al.*^2014^). In addition, some host proteins, including heparan sulfate, integrin, ephrin A2, xCT and dendritic cell-specific ICAM-3 grabbing nonintegrin (DC-SIGN), can serve as receptors for KSHV and facilitate attachment of the virion to the cell membrane (Akula *et al.*^2002^; Hahn *et al.*^2012^; Hensler *et al.*^2014^; Kaleeba and Berger 2006; Rappocciolo *et al.*^2006^). KSHV primary infection outcome and gene expression after infection vary in different target cells, such as endothelial, epithelial, B cells, keratinocytes and monocytes, which predominantly establish persistent latent infection and express mainly the latent transcriptome (Jha *et al.*^2014^; Purushothaman *et al.*^2015^; Singh *et al.*^2014^). Specifically, infection of primary tonsillar lymphocytes with KSHV causes an abortive lytic infection of T cells (Myoung and Ganem 2011b). Therefore, latency is the default life cycle for KSHV following host cell infections (Giffin and Damania 2014).

**Fig. 2:**
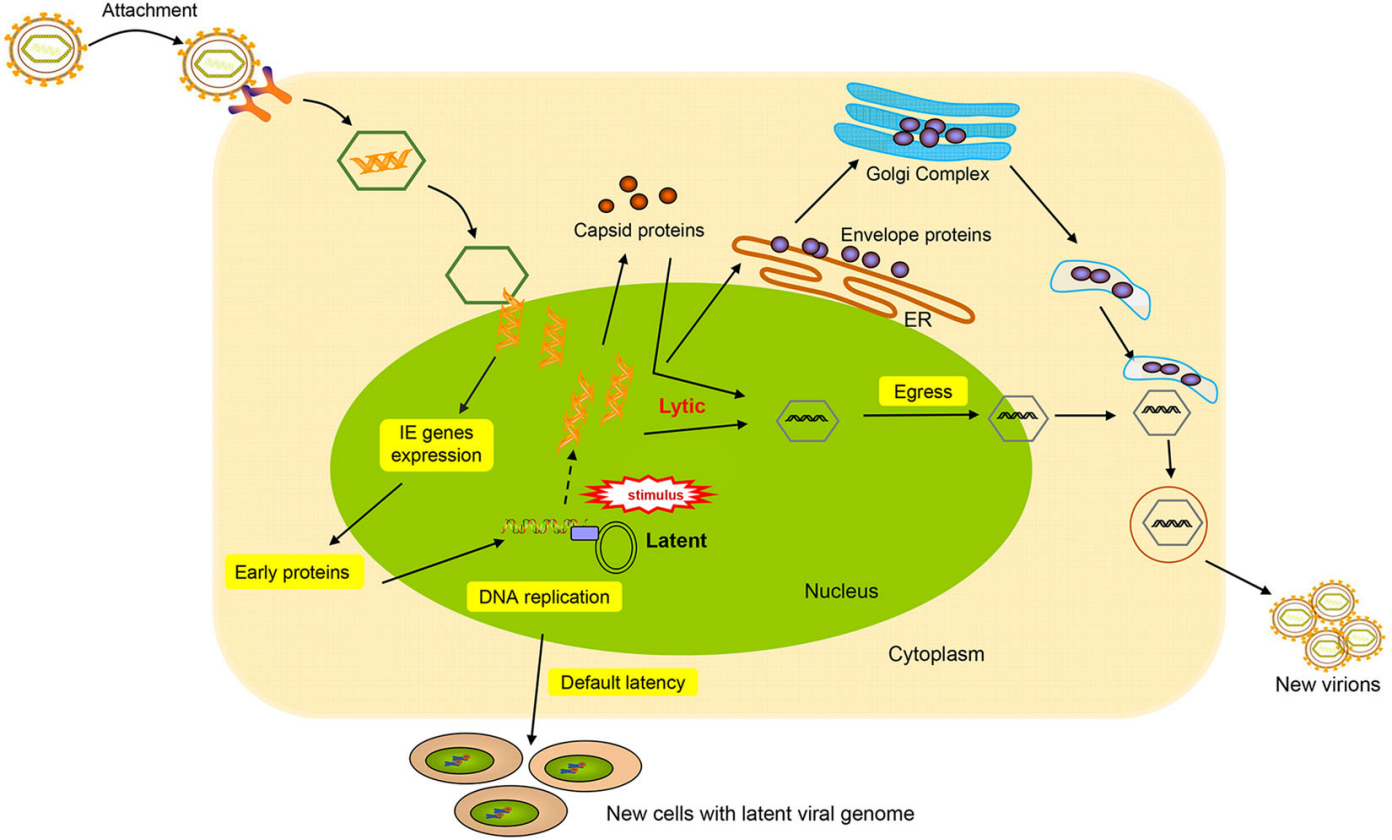
Diagram of KSHV life cycle events. Depicted are virion attachment, entry into the host cells and the different life cycles decision. KSHV entry is a multiple step involved in viral glycoproteins and many cellular membrane proteins. The entry is initiated by the binding of glycoproteins to specific cell receptors in the host cell membrane. After the entry, KSHV genome packaged in capsid is transported through the cytosol and then the genome is released into the nucleus. Once the viral genome enters the nucleus, the decision to enter the latent or lytic phase of the life cycle is made. KSHV life cycle contains two phases of infection: a short lytic replication and a persistent latent replication. During latency, LANA protein tethers KSHV episome to host cell chromosome and partitions certain numbers of genome copies to new cells. Upon exogenous stimuli, KSHV can be induced to lytic cycle. During lytic cycle, the KSHV genome replicates in a rolling cycle mechanism to produce linear genomes that are packaged into capsids. Mature capsids then obtain virus envelop by budding on host membranes. Lytic replication subsequently leads to destruction of the cells and release of virions to infect new cells.

Similar to other members of the herpesvirus family, KSHV displays two different modes of infection, latent infection and lytic reactivation, differentiated by complex but characteristic gene expression patterns (Cai *et al.*^2010^; Ye *et al.*2011a). During latency, the viral genome circularizes in the nucleus and maintains as a chromatinized nuclear plasmid; thus, no virion production occurs, and only a small subset of genes are expressed (Ballestas *et al.*^1999^). Latent DNA replication proceeds from multiple origins of replication (*ori*-*P)* in the TRs with the aid of host cellular DNA polymerase and a limited genome copy number is maintained via even distribution to daughter cells (Deng *et al.*^2012^; Lin *et al.*^2003^). The latent state is not invariable and can be disrupted by environmental and physiological stimuli, such as oxidative stress and hypoxia (Davis *et al.*^2001^; Ye *et al.*2011b). The switch may activate the resting genome, which features a substantial and sequential viral gene expression and viral DNA replication (Chen *et al.*^2001^; Haque *et al.*^2003^). During reactivation, lytic genes begin to be expressed in a temporally regulated transcriptional cascade as follows: (1) immediately early (IE) genes, which primarily encode transcription factors and regulators; (2) early genes, which function in preparation of the cell ready for viral DNA replication and viral protein production; and (3) late genes, which are subsequent to the onset of replication and comprise viral structural components (Carroll *et al.*^2007^; Davis *et al.*^2015^; Jenner *et al.*^2001^; Saveliev *et al.*^2002^; Zhu *et al.*^1999^). After all classes of the lytic genes are expressed, virus assembly begins in the nucleus. The replicated genomes are incorporated into newly synthesized capsids, acquire teguments, and finally bud through host membranes to obtain envelopes. Subsequently, viral progeny are released from the host cell (Gradoville *et al.*^2000^). The boundary between the latent and lytic state is not clear. In the KSHV lytic infection, virus-infected cells display both lytic and latent gene expression simultaneously. A brief description of KSHV life cycles is summarized in Fig. 2.

## Regulation of Viral Latency

### Establishment of KSHV Latency

In terms of viral genome persistence in infected cells, KSHV has developed many mechanisms to establish long-term, nonproductive latent infection. To accomplish this goal after lytic infection, the viral genome has to first be circularized and chromatinized into a new form of epigenome to tightly control early gene expression in an off–on latent-specific pattern (Ballestas and Kaye 2001). Although the transcriptional pattern exhibits latency, the virus genome maintains the potential to enter the primary infection phase. Thus, suppression or silencing of viral lytic gene expression is the mechanism by which KSHV remains latent. Furthermore, during the centuries’ coevolution of the virus and host, KSHV has acquired a number of genes that selectively evade host immune system to maintain its persistent infection (Areste and Blackbourn 2009; Coscoy 2007; Lee *et al.*^2010^, ^2012^, 2016a).

### Transcription Regulation of Latent Genes

Only a small portion of the latent KSHV genome is transcribed, and the major latency locus in latently infected PEL cells includes the genes for ORF73 (latency-associated nuclear antigen, LANA), ORF72 (v-Cyclin), ORF71 (K13) (*Fas*-*associated death domain*-*like interleukin*-*1β*-*converting enzyme* (FLICE)-*inhibitory protein,* v-FLIP), and K12/Kaposin (Kaposin A, B and C) and about 25 mature microRNAs (miRNAs) (Cai and Cullen 2006; Cai *et al.*^2005^; Dittmer *et al.*^1998^; Gottwein *et al.*^2006^, ^2011^; Kedes *et al.*^1997^; Staskus *et al.*^1997^). These genes, positioned in the minus strand of double-stranded viral genome, are transcribed from different latency promoters; LANA, v-Cyclin and v-FLIP are transcribed from the LANA promoter or LT_c_, while the others are transcribed from the Kaposin promoter, or LT_d_ (Pearce *et al.*^2005^; Sadler *et al.*^1999^). In KSHV-infected MCD and PEL tissues, another latency locus encoding the vIRF3 gene was found to be expressed as the LANA-2 (K10.5), which is known to inhibit interferon induction and thus enhance cell proliferation and survival (Fakhari and Dittmer 2002). In addition, using array-based transcript profiling with limiting-dilution reverse transcription-PCR (RT-PCR), ORF K1 had been found to be weakly transcribed in most latently-infected cells, but remains upregulated during lytic reactivation (Chandriani and Ganem 2010). Similarly, examination of KS and PEL tumors also showed that several other viral genes including ORF74, K14, vIL-6 and ORF59, are transcribed at low levels (Majerciak *et al.*^2013^; Wen and Damania 2010), indicating a certain degree of the leaky expression of viral lytic genes during latent infection.

#### ORF73 (LANA)

LANA, the most abundant latent protein in both latently-infected cells and tumors, is about 1162 amino acids in length. The N-terminal region of LANA mainly associates with many chromatin-associated proteins, while the C-terminal region connects to numerous cellular proteins and chromosomes. The middle part of the protein contains a large repetitive region composed of acidic and glutamine-rich repeats resulting variable size of LANA protein (Ballestas and Kaye 2001; De Leon Vazquez and Kaye 2011; Kelley-Clarke *et al.*^2009^; Uppal *et al.*^2014^).

To maintain viral genome stability and replication, the C-terminus of LANA binds to the conserved TR repeats of the KSHV genome directly, while the N-terminal domain then loads the viral genome on host genome via interacting with chromatin proteins, thus avoiding genome loss during cell mitosis and maintaining a relatively low number of viral DNA copies in infected cells. Several cellular proteins have been reported to interact with LANA, including ATF4/CREB2/STAT3 for transcription, p53/pRb/von Hippel Lindau (VHL) for tumor repression, HP1/H2A/H2B/MeCP2/BRD4 for chromatin binding, and GSK-3β for signal transduction (Cai *et al.*^2006^; Fujimuro *et al.*^2005^; Garber *et al.*^2001^; Griffiths and Whitehouse 2007; Jr *et al*. 1999; Lim *et al.*^2000^; Ottinger *et al.*^2006^; Radkov *et al.*^2000^). LANA also directly binds to many epigenetic regulatory proteins, including DEK, nucleophosmin (NPM), CENP-F, DNMT3a, TRF1, topoisomerase II β (TopoII β), TIP60 and KDM3A (Krithivas *et al.*^2002^; Ottinger *et al.*^2006^; Purushothaman *et al.*^2012^; Sarek *et al.*^2010^; Shamay *et al.*^2012^; Xiao *et al.*^2010^). LANA function can also be regulated by post-translationally modifications of the protein. For example, PRMT1 mediates LANA lysine acetylation and arginine methylation, while Pim-1, CK1, GSK1, GSK3 and RSK3 lead to LANA phosphorylation (Bajaj *et al.*^2006^; Campbell *et al.*^2012^; Cheng *et al.*^2009^; Lu *et al.*^2006^; Woodard *et al.*^2012^). As reported, LANA binds to several viral lytic promoters to inhibit viral lytic gene transcription, which is an important mechanism for the maintenance of latency (Hu *et al.*^2014^). For example, LANA prevents RTA-mediated activation of lytic replication by competitive interaction with RBP-Jκ (Lan *et al.*^2004^, 2005a).

#### ORF72 (v-Cyclin)

ORF72 is the homolog of cellular cyclin D2 and similarly functions in regulating the cell cycle and cell proliferation by constitutive activation of cellular cyclin-dependent kinase 6 (CDK6) (Jones *et al.*^2014^; Li *et al.*^1997^; Van Dross *et al.*^2005^). The vCyclin-CDK6 complex was found to have a large range of substrates and phosphorylates cellular p21 and p27, resulting in accelerated G_1_/S phase transition in PEL cell lines (Chang and Li 2008; Godden-Kent *et al.*^1997^; Jarviluoma *et al.*^2004^; Laman *et al.*^2001^). In addition, nucleophosmin (NPM) was found to be phosphorylated by the complex to control latency program in KSHV-infected cells (Sarek *et al.*^2010^). Deletion of v-Cyclin with the homologous recombination method was found to affect cell proliferation and cell cycle progression in a density-dependent manner (Jones *et al.*^2014^).

#### ORF71 (K13, v-FLIP)

ORF71 or v-FLIP is a homolog of cellular FLICE inhibitory protein (Krueger *et al.*^2001^; Li *et al.*^2006^). The best characterized function of ORF71 is to activate a key cellular signaling pathway, NF-κB pathway, by directly binding to the IκB kinase γ (IKKγ) complex and thus facilitate cell survival, proliferation and cell type-specific induced growth arrest and apoptosis during latency (Bagneris *et al.*^2008^; Chaudhary *et al.*^1999^; Field *et al.*^2003^; Graham *et al.*^2013^; Grossmann *et al.*^2006^; Matta *et al.*^2007^). Furthermore, v-FLIP suppresses the AP-1 pathway by activating NF-κB pathway to inhibit viral lytic replication and promote latency (Ye *et al.*^2008^). In addition, many cellular proteins involved in the activation of NF-κB pathway are induced by v-FLIP via protein–protein interaction, such as cell adhesion molecule 1 (CADM1) and NEMO (Hunte *et al.*^2018^; Matta *et al.*^2012^). Recently, the v-FLIP coding sequence-deleted virus has been constructed to investigate more function of this gene in KSHV life cycles (Wang *et al.*^2018^).

#### Kaposins (K12)

In contrast to the three latent genes described above, the Kaposin locus is located downstream of the LANA promoter and encodes a complex of proteins, including Kaposins A, B and C (Sadler *et al.*^1999^; Sarid *et al.*^1999^). Kaposin A, a hydrophobic polypeptide, is mainly found on intracellular and cell surface membranes (Tomkowicz *et al.*^2002^). As the smallest isoform from the K12 locus, Kaposin A can induce focal transformation *in vitro* in Rat-3 fibroblasts and nude mice, indicating its oncogenic potentials in induction of the transformation phenotype (Muralidhar *et al.*^1998^). Kaposin A interacts with the guanine nucleotide exchange factor cytohesin-1 and activates ERK2/MAPK (Kliche *et al.*^2001^), while Kaposin B enhances the expression of cytokines by inhibiting the degradation of their mRNAs containing AU-rich elements (AREs) in their 3′ noncoding regions via binding to and activating p38/MK2 pathway (McCormick and Ganem 2005). In addition, Kaposin B was recently found to regulate microRNAs cooperated with c-Myc in KSHV-infected cells (Chang *et al.*^2016^; Corcoran and McCormick 2015). To date, no published studies have examined Kaposin C (Speck and Ganem 2010).

#### Viral miRNAs

miRNAs, a group of noncoding single-stranded RNAs approximately 19–23 nucleotides (nt) in length, primarily regulate gene expression at the posttranscriptional level by binding to the seed-matched regions of target mRNAs (Ameres and Zamore 2013). KSHV has been identified to encode 25 mature viral miRNAs from 12 viral pre-miRNAs (Cai *et al.*^2005^; Grundhoff *et al.*^2006^; Pfeffer *et al.*^2005^). Mature miRNAs are abundantly expressed in latently infected cells and tissues from patients with KS or MCD (Grundhoff *et al.*^2006^; Sullivan 2007), and according to published studies, some specific KSHV miRNAs were regulated at different levels in different phases of the viral life cycle (Qin *et al.*^2017^). Some miRNAs were also detected in KSHV virions and function in cellular communication, while a few miRNAs from patient exosomes were found to enhance cell migration (Chugh *et al.*^2013^; Lin *et al.*^2012^).

As reported, KSHV miRNAs function in regulating virus life cycles, cell immune response, virus-induced angiogenesis and spreading of KS by targeting and inhibiting the expression of multiple cellular and viral genes. For instance, miR-K9-5p and miR-K12-7 together with miR-K12-9 were identified to inhibit RTA expression, resulting in maintenance of viral latency (Bellare and Ganem 2009; Lin *et al.*^2011^). Other miRNAs like miR-K11 and miR-K3, also target cellular factors, such as IκB α, BCLAF1 and nuclear factor (I/B) to promote latency (Lei *et al.*^2010^; Lu *et al.*^2010^; Ziegelbauer *et al.*^2009^). A role of miR-K3 in maintaining viral latency is to directly target G protein-coupled receptor kinase 2 (GRK2) to activate CXCR2/AKT signaling and promote endothelial cell migration and invasion (Hu *et al.*^2015^; Li *et al.*2016b). In addition, at least two KSHV miRNAs were found to functionally mimic cellular miRNAs by targeting same transcripts, including miR-K12-11 mimicking hsa-miR-155 (Gottwein *et al.*^2007^; Skalsky *et al.*^2007^) and miR-K10a mimicking hsa-miR-142 (Forte *et al.*^2015^). KSHV miRNAs also manipulate host immune surveillance to promote latency. For instance, miR-K12-11 controlled the IFN signaling by targeting IKKε to suppress antiviral immunity, resulting in establishment of long-term latency (Liang *et al.*^2011^). miR-K12-7 targets the stress-induced immune molecule (MICB) during virus infection to avoid the attacks by natural killer (NK) cells (Nachmani *et al.*^2009^). miR-K10a targets tumor necrosis factor-like weak inducer of apoptosis receptor protein (TWEAKR) and the inhibition prevents TWEAK-induced apoptosis and inflammatory cytokine (IL8) expression (Abend *et al.*^2010^). miR-K9 and miR-K5 target the 3′UTR of interleukin-1 receptor (IL-1R)-associated kinase (IRAK1) and myeloid differentiation primary response protein 88 (MYD88) respectively, the overexpression of the miRNAs leads to the reduction of secreted IL-6 and IL8 in HUVEC cell culture (Abend *et al.*^2012^).

KSHV miRNAs also play important roles in KS angiogenesis and development. miR-K2 and miR-K5 target and reduce the different isoforms of tumor suppressor protein tropomyosin 1 (TPM1), which both lead to enhanced tube formation and VEGFA expression critical to KSHV angiogenesis and pathogenesis (Kieffer-Kwon *et al.*^2015^). Breakpoint cluster region mRNA (BCR) was also identified as a target of miR-K6-5, and the suppression increased the tube formation in HUVECs transfected with miR-K6-5 *in vitro* and lytic reactivation in BCBL-1 cells (Ramalingam *et al.*^2015^). miR-K6-3p was found to promote endothelial cell migration and angiogenesis by targeting 3′ UTR of SH3 domain binding glutamate-rich protein (SH3BGR) and activating STAT3 pathway (Li *et al.*2016c). Recently, miR-K6-5p was found to target metastasis suppressor CD82 while inhibition increased the cell invasion and angiogenesis by activating c-Met signaling (Li *et al.*^2017^).

### Suppression of Viral Lytic Genes

Broad repression of viral lytic gene expression is one of the mechanisms by which latency is established and maintained. After *de novo* infection, the viral genome has to be first chromatinized, and the viral LANA protein then recruits the host polycomb repressive complexes (PRC1 and PRC2) to the promoters of lytic genes to initiate latency establishment (Toth *et al.*2013b, 2016). Depletion of the LANA protein in the context of the KSHV genome decreased the recruitment of PRCs to the viral genome during infection. Therefore, LANA can function as a genome-wide repressor of lytic gene expression. Studies have also reported that LANA directly binds to and inhibits RTA promoter activities. Moreover, LANA indirectly and directly binds to other transcription activators and repressors, such as Sp1, RBP-Jκ and KAP1, all of which can regulate the expression of RTA (Lan *et al.*^2004^, 2005a; Sun *et al.*2014b; Verma *et al.*^2004^). Experiments performed using small interfering RNA in PEL cells or using the LANA deletion mutant BAC36ΔLANA showed that LANA could repress the viral lytic reactivation phase, as it repressed the expression of all classes of viral lytic genes and the final production of infectious KSHV virions (Li *et al.*^2008^).

### Epigenetic Regulation of Latency

Like other herpesvirus members, KSHV latency is governed in part by epigenetic modifications. To maintain the quiescent state of limited latent gene expression, some critical lytic genes are silenced, but treatment of latently infected PEL cells with DNA methyltransferases and histone deacetylases inhibitors induces lytic reactivation and replication (Miller *et al.*^2007^). Epigenetic modifications of the herpesvirus genome are mainly characterized as three groups: DNA methylations, chromatin protein posttranslational modifications and higher-order chromosome conformations (Chen *et al.*^2013^).

#### DNA Methylation

DNA methylation patterns are important for regulating KSHV latency (Gunther and Grundhoff 2010), and DNA methylation is typically indicative of repressed transcription and stabilized viral latency. To explore the potential roles of such modifications of KSHV gene expression, initial studies focused on the promoters of ORF50 and ORF73/LANA, two key molecules during the KSHV life cycle. However, subsequent studies on KSHV-positive tumor samples did not yield similar results, as the RTA promoter was heavily methylated in BCBL-1 cells, while the LANA promoter was not. Furthermore, the global methylation status of viral episomes has been examined in PEL derived cell lines using MeDIP (methylated DNA immunoprecipitation) technology, revealing that DNA in the KSHV genomes is profoundly methylated during latency (Gunther and Grundhoff 2010). The genome mainly includes regions upstream of the LANA promoter (LANAp) and other locations, including K7, K9, ORF45/50 and ORF8 (Gunther and Grundhoff 2010).

#### Histone Modifications

Histone modifications have also proven to be indispensable for regulating KSHV latency. These markers include the active histone acetylation markers histone H3 and H4N-terminal tails (e.g. H3K9/K14-ac) and trimethylation of H3 at lysine 4 (H3K4-me3) (Lu *et al.*^2003^) as well as, the repressive histone modifications of lysine 9 trimethylation in histone H3 (H3K9-me3), a hallmark of constitutive heterochromatin, and trimethylation of lysine 27 (H3K27-me3).

High-resolution genome-wide studies on histone modifications during latency and reactivation have been conducted, revealing a distinctive pattern of activating and repressing markers during latency and inducing changes upon reactivation. The latency-associated locus was shown being enriched by activating the H3K4-me3 and AcH3 histone modifications, but no repressive H3K9-me3 and H3K27-me3 markers. DZNep, a small molecule inhibitor of the H3K27-me3 methylase, was shown to stimulate KSHV lytic cycle gene activation, suggesting a positive role of H3K27-me3 in latency establishment and maintenance (Bernstein *et al.*^2006^). The promoter regions of RTA and other early genes are associated with both activating (H3K4-me3) and repressing (H3K27-me3) histone modifications, which indicates multiple and dynamic mechanisms of regulating chromatin structure during viral gene expression (Toth *et al.*^2010^, 2013a). In terms of latency maintenance, histone modifications function similarly to the on–off switch of the latent state and reactivation phase by selectively expressing latent and lytic loci in the KSHV genome (Toth *et al.*2013b).

As noted above, histone modifications have profound impacts on the existence of the virus in cells, and the factors responsible for managing histone modifications and nucleosome positioning may thus be substantially relevant for understanding the epigenetic control of KSHV latency. CCCTC-binding factor (CTCF) is known to function as a chromatin-organizing factor (Dorsett 2011; Herold *et al.*^2012^; Ohlsson *et al.*^2001^; Van Bortle and Corces 2013a). However, a study on the KSHV genome-wide histone modification following *de novo* infection of SLK cells indicated an increased H3K27-me3 for K12 but no change for LANA, whereas the viral genome exhibited a remarkable increase of H3K4-me3 for both K12 and LANA in the course (4–72 h) of KSHV infection. Unfortunately, the heterochromatin hallmark H3K9-me3 on these protected sites, such as the KSHV LANA promoter region was not included in this study (Toth *et al.*2013b), raising the question about their biological significance of the reported histone modifications in the lytic-to-latent switch. Whether the chromatin boundary element prevents the protected regions from being occupied by repressive markers needs to be uncovered. The impaired episome stability after deletion of CTCF from the LANA promoter region confirmed this speculation (Chen *et al.*^2012^), but whether this loss is associated with an increase in H3K9-me3 or DNA methylation need to be further explored.

#### Chromatin Conformation

Higher-order chromatin structures, such as those resulting from promoter-enhancer DNA loop interactions, have been found on KSHV chromatin to influence the coordinated control of gene expression (Kang H *et al*. 2011; Van Bortle and Corces 2013b). A DNA loop with CTCF-cohesin binding sites in the LANA promoter region was found to interact with the ORF50 promoter region and regulate the lytic gene expression (Kang H *et al*. 2011). Disruption of the DNA loop stability, which mainly depends on cellular factors involved in sister-chromatid cohesion, led to reactivation of the lytic cycle, suggesting the importance of this chromatin conformation in maintaining latency (Chen *et al.*^2012^).

### Mechanism of KSHV Latent Replication

To maintain the potential of the silenced virus genome during latency, KSHV has evolved its own strategy to simultaneously replicate the viral genome and cellular DNA. Furthermore, the replicated genome has to be precisely passed to daughter cells and keep effectively maintained at 10–150 genome copies per infected cell (Li *et al.*^1996^; Renne *et al.*1996a; Sturzl *et al.*^1997^; Wen and Damania 2010).

DNA replication is a multistep and sequential event that begins with the assembly of pre-replication complex (pre-RC) proteins at the replication origin. Generally, DNA replication is initiated at many replication origins almost simultaneously such that the process is completed within a limited time frame. Replication of the KSHV viral genome has been shown to initiate at multiple points associated with the TR region that contains an origin of replication, *ori*-*P* (Verma *et al.*^2011^). LANA can directly bind to *ori*-*P* via LANA-binding sites (LBS-1/2) and a 32-bp GC-rich segment (Hu and Renne 2005; Ohsaki and Ueda 2012; Verma *et al.*2007a), thus triggering the recruitment of various (pre-RC) proteins to *ori*-*P*. The pre-RC contains at least the origin recognition complex (ORC), poly(ADP-ribose) polymerase 1 (PARP1) and minichromosome maintenance proteins (MCM) within its nuclear matrix region (Lim *et al.*^2002^; Ohsaki *et al.*^2004^; Stedman *et al.*^2004^). Interestingly, LANA also interacts with and recruits cellular machinery to support latent replication. The cellular machinery recruited by LANA includes bromodomain containing 2 (BRD2), origin recognition complex 2 (ORC2), H4-specific histone acetylase (HBO1) and CREB-binding protein (CBP) (Stedman *et al.*^2004^), and the interactions occur in the carboxy-terminus of LANA, i.e., the TR binding domain (DBD). Subsequent to the recruitment of ORCs to the replication origin, some DNA replication licensing factors, such as MCMs, are directed to the same sites for replication (Tsuyama *et al.*^2005^).

LANA also interacts with replication factor C (RFC) ATPase, resulting in the augmentation of PCNA loading onto the DNA to facilitate the replication and persistence of viral DNA (Sun *et al.*2014a). PCNA is identified as a DNA sliding clamp that increases the proximity of replicative DNA polymerase for better processivity and is an essential component of the DNA replication machinery (Moldovan *et al.*^2007^). In addition, BUB1, a cellular mitotic kinase, was found to enhance the monoubiquitination of PCNA in the presence of LANA, which contributes to episome replication and maintenance (Sun *et al.*^2015^). Further studies have shown that LANA also interacts with and recruits cellular DNA topoisomerase 2-β (TopoII β) to the TR region. The main function of TopoII β protein is regulating and modifying the DNA topology by introducing double-stranded breaks, and thus plays a critical role in viral DNA replication (Purushothaman *et al.*^2012^).

In addition to *ori*-*P*, *ori*-*A* has been identified as another initiation site of latent DNA replication that is independent of LANA (Verma *et al.*2007b, 2011). Single molecule analysis of replicated DNA (SMARD) of the entire KSHV genome verified that more than one site in the genome can initiate replication. Chromatin immunoprecipitation (ChIP) experiments to detect ORC2 and MCM3 accumulation suggested that various regions of the genome might potentially serve as the replication sites (Verma *et al.*^2011^).

## Lytic Reactivation

The latent state of KSHV infection is reversible and inducible. Once the latency is disrupted by physiological factors or an exogenous stimulus, the virus begins the new phase of its life, lytic reactivation, by consecutively expressing most viral genes, i.e., immediate early, early and late genes, finally resulting in the production of infectious virion particles.

As a substantial number of viral genes are expressed, viral DNA is amplified, and the virus simultaneously exploits the cellular machinery to generate new infectious virions that are released from the infected cells to spread the virions to other susceptible cells. To better promote survival and reproduction, KSHV does not kill the resident host. Furthermore, as latent viral infection is the outcome occurring most often, the biological significance of the virus having both a latent and a lytic cycle are obvious: latent phase for maintaining the silenced virus genome for long-term, but producing no viruses, but lytic phase for virus propagation and spreading the virus to a new host. The lytic cycle may be also important for virus-mediated disease development. The viral load in peripheral blood mononuclear cells in patients correlates with KS progression (Quinlivan *et al.*^2002^). To control the KSHV-mediated malignancies in KS patients and AIDS individuals who are susceptible to KS, some drugs that block viral lytic DNA replication, such as foscarnet and ganciclovir, have been proven to repress early KS. In most KS lesions, the tumor-forming spindle cells are latently infected, but a small percentage of cells still undergo spontaneous lytic replication. Collectively, the lytic cycle not only plays an important role in the intact virus life cycle for virus propagation, but also has indispensable functions in KSHV pathogenicity.

Various host and experimental factors trigger virus reactivation from latently infected cells. To investigate the mechanism underlying the switch between the latent and reactivation states, several chemical reagents, including 12-O-tetradecanoyl-phorbol-13-acetate (TPA) (Miller *et al.*^1997^), sodium butyrate, valproic acid, ionomycin, and 5-azacytidine, have been widely used (Al-Kzayer *et al.*^2016^; Lukac *et al.*1999b; Shin *et al.*^2014^). While KSHV infection is necessary for the development of KSHV-associated diseases, it is not sufficient to induce tumorigenesis (Andrei and Snoeck 2015; Ganem 2010). Several infectious pathogens known to occur simultaneously with KSHV-associated oncogenesis include HIV, Epstein-Barr virus (EBV), human cytomegalovirus (HCMV), human herpesvirus-6 and -7 (HHV-6 & -7), herpes simplex virus type 1 and type 2 (HSV-1 & -2) and human papillomaviruses (Adams *et al.*^1995^; Tang *et al.*^2012^). Reports conducted in PEL cells have proven that hypoxia may contribute to KSHV lytic reactivation (Davis *et al.*^2001^). In addition, oxidative inhibition can be a strategy for inhibiting viral replication and tumorigenesis, as treatment with antioxidant/H_2_O_2_ scavengers inhibits KSHV lytic replication and tumor progression *in vivo* (Ye *et al.*2011b). After initiation of the virus lytic cycle, many viral proteins and cellular factors are involved in regulation of the lytic events.

### Viral Proteins with Transactivation Functions

#### ORF50 (Replication and Transcription Activator, RTA)

RTA plays a major role in KSHV lytic reactivation from viral latency. RTA mRNA is about 3.4 kb in length and has seven spliced isoforms. Because RTA utilizes a poly A signal downstream of K8.1 for its RNA polyadenylation, its 3′UTR region overlaps with K8 and K8.1 region (Tang and Zheng 2002; Zheng 2003). RTA can be detected within 4 h of primary infection (Purushothaman *et al.*^2015^) and in TPA-induced BCBL-1 (PEL) cells (Dourmishev *et al.*^2003^; Sun *et al.*^1999^). As a transcription factor, RTA is localized in the nucleus and contains two nuclear localization signals (NLSs).

RTA itself is essential and sufficient for KSHV reactivation (Lukac *et al.*1999a). KSHV genome with RTA deletion is defective in expression of early lytic viral genes, virus production and DNA synthesis. Many studies have demonstrated the RTA transactivates a series of important lytic genes, including viral early genes (such as PAN, thymidine kinase [TK or ORF21], vIL-6 [K2], ORF57, ORF59, K8, K9, K1, DBP [ORF6], and DNA polymerase [ORF9]) and the late genes, assembly protein (AP) and gB (ORF8) (Byun *et al.*^2002^; Chen *et al.*^2009^). RTA also transactivates the promoters of ORF52, ORF65, ORF56, ORF37 and vOX (K14) (Bu *et al.*^2008^; Chen *et al.*^2009^), and autoactivates its own promoter (Deng *et al.*^2000^). RTA transactivates viral promoters by two mechanisms: by directly binding to the RTA response elements (RREs) in the promoter, and by indirectly binding to the RREs with other cellular or viral proteins, such as the Notch signaling pathway effector recombination signal binding protein (RBP-Jκ) (Persson and Wilson 2010). Other cellular transcription factors involved in RTA transactivation activity are C/EBPα (CAAT/enhancer binding protein alpha), Oct-1 (octamer-binding protein-1), Sp-1, and XBP-1 (Carroll *et al.*^2007^; Lai *et al.*^2011^; Wang *et al.*^2003^; Wen *et al.*^2009^).

In addition to its transactivation ability, RTA also exhibits an ubiquitin E3 ligase activity, allowing cellular and viral proteins to undergo proteasome-mediated degradation. MYD88, a key adaptor of innate immunity to microbial infection, interacts with RTA *in vitro* and *in vivo*, and is a target of RTA E3 ligase activity, thus helping the virus evade innate immunity (Zhao *et al.*^2015^). RTA can be phosphorylated, ADP-ribosylated, ubiquitinated and modified into other forms (Campbell and Izumiya 2012; Ko *et al.*^2012^; Tsai *et al.*^2012^).

Apart from transcriptional regulation and proteasome-mediated degradation, RTA also activates lytic DNA replication by binding to C/EBPα and the RRE-binding motifs within the origin of lytic replication (*ori*-Lyt) for transcriptional activation and recruitment of additional factors.

#### K8 (Lytic Replication-Associated Protein)

The K8 encodes a protein designated as K-bZIP (K8α), belonging to the basic-leucine zipper (bZIP) family of transcription factors. The K-bZIP gene locus contains two promoters, one for early transcribed product K-bZIP, and one for late transcribed product K8.1 (Tang and Zheng, 2002). K-bZIP was discovered to interact with RTA and, in turn, repress the transactivation activity of RTA, which seems to play a negative role in viral lytic gene expression and replication (Liao *et al.*^2003^). K-bZIP induces p53 and p21 expression, directly interacts with CDK2 and promotes cell cycle arrest (Izumiya *et al.*^2003^). However, the function of K-bZIP could be antagonized by K8β, a truncated K-bZIP translated from a spliced K8 mRNA isoform (Yamanegi *et al.*^2005^). A recent study also showed that K-bZIP is not essential for lytic reactivation in KSHV BACmid systems, but is important for virus production in infected PEL cells (Wang *et al.*^2011^).

K-bZIP is associated with *ori*-*Lyt*-dependent DNA replication via its interaction with *ori*-*Lyt* (Lin *et al.*^2003^). In addition, K-bZIP interacts with HDAC to inhibit the recruitment of HDAC to the promoters of *ori*-*Lyt* and ORF50, thus repressing the RTA autoactivation (Martinez and Tang 2012). K-bZIP mediated transcription repression is, in part, associated with sumoylation at lysine residue 158. A genome-wide analysis showed that K-bZIP activated 21 promoters, while RTA activated 34 viral promoters (Ellison *et al.*^2009^). When combined, K-bZIP appears to repress three RTA-responsive promoters, suggesting additional role of K-bZIP in transactivating viral promoters during reactivation (Liao *et al.*^2003^). Collectively, K-bZIP has dual functions in inhibiting lytic gene expression under certain conditions and regulating the KSHV life cycle by promoting lytic DNA replication. Although not binding DNA, K8 binds T1.4 RNA to recruit viral DNA replication factors to *ori*-*Lyt* DNA and regulates KSHV DNA replication (Liu *et al.*^2018^).

### Cellular Factors

**Fig. 3:**
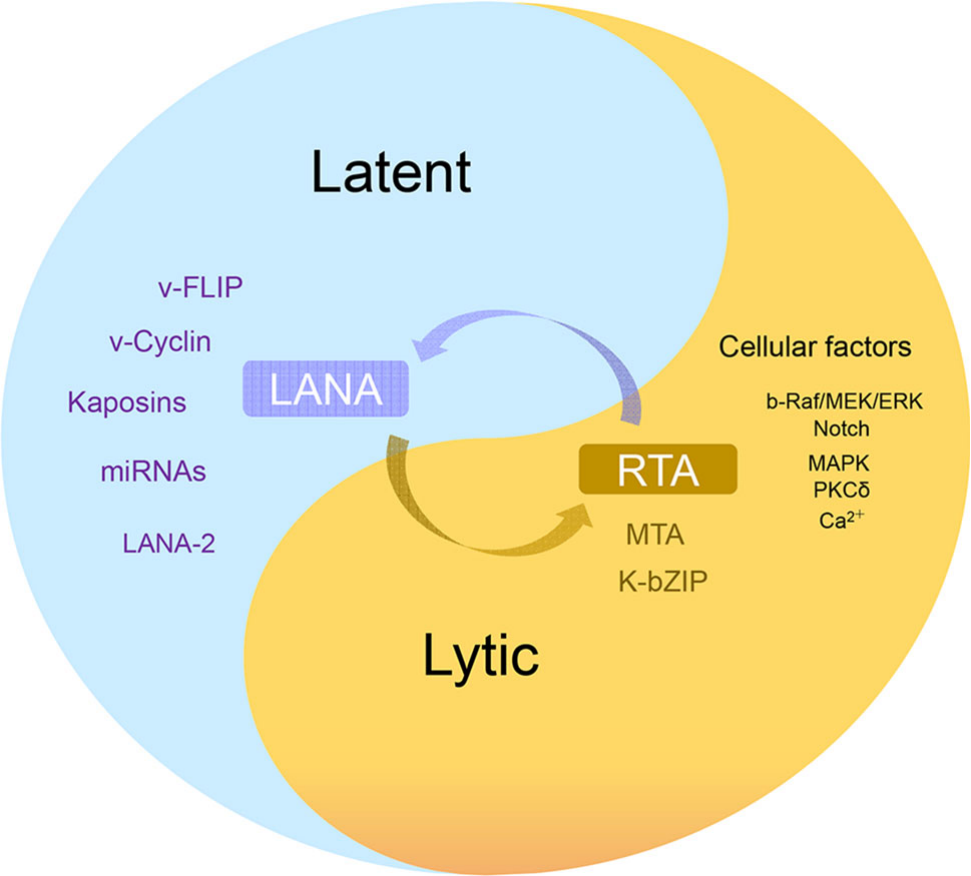
Regulation of KSHV latency and lytic reactivation. The purple are viral latent proteins, the brown are viral critical lytic proteins, the black is a few of cellular factors and signaling pathways involved in viral lytic cycle.

Several cellular signaling pathways are involved in KSHV reactivation in latently infected cells, including PKC δ, PKA, b-Raf/MEK, p38 and JNK, Notch and RBP-Jκ, PI3K and Akt, TLR7/8 signaling and Pim-1/3 (Ford *et al.*^2006^; Gregory *et al.*^2009^; Haas *et al.*^2013^; Lan *et al.*^2006^; Xie *et al.*^2005^; Yu *et al.*^2007^). Protein kinase C δ plays a role in KSHV lytic replication, as overexpression of the dominant-negative PKC δ mutant supports an essential role for the PKC δ isoform in virus reactivation. Using specific pathway-targeted inhibitors, studies have found that b-Raf/MEK/ERK signaling plays a role in TPA-induced reactivation from latency. The JNK and p38/MAPK pathways were constitutively activated in latent BCBL-1 cells, but TPA treatment increased the levels of only activated ERK and p38 and reduced the expression of viral lytic genes, including RTA. Activation of MAPK pathways was shown to be necessary for activating the RTA promoter. After screening the human protein kinase cDNA library and analysis of complex cross-talk between cellular kinase pathways, Pim-1 and Pim-3 were identified as novel kinases involved in KSHV reactivation. Phosphorylation of LANA by Pim-1 and Pim-3 counteracts the LANA-dependent repression of viral transcription (Bajaj *et al.*^2006^; Cheng *et al.*^2009^). Similarly, screening involving TLR signaling in reactivation showed that TLR7/8 agonists reactivated latent KSHV and induced viral lytic gene transcription and replication. Apart from the above signaling pathways, ionomycin, a Ca^2+^ ionophore, was shown to induce RTA protein expression, which suggests a role of calcium-dependent signaling pathways in virus reactivation (Zoetweij *et al.*^2000^) (Fig. 3).

## Posttranscriptional Regulation of Viral Gene Expression

Beside the regulation of transcription, the eukaryotic cells developed several additional mechanisms to regulate gene expression after transcription. As these processes occur after transcription, they are commonly referred as posttranscriptional and involve many cellular RNA-binding proteins and small RNAs. The first layer of such regulation comprises of proper processing of nascent transcripts including RNA capping, splicing, and polyadenylation to produce mature mRNA. Nuclear export of the mature mRNA to the cytoplasm and mRNA translation and turnover are the additional layers of posttranscriptional regulation of eukaryotic gene expression (Schaefke *et al.*^2018^). Recently, several groups found the newly transcribed viral RNA also harbors post-transcriptional N^6^-adenosine methylation marks and this event plays important roles in viral lytic replication based on the observation that some m^6^A sites are responsible for RTA pre-mRNA splicing and RTA increases its own expression through posttranscriptional mechanism (Ye 2017; Ye *et al.*^2017^). Further, the m^6^A epitranscriptome of KSHV had been mapped in diverse cell types both in latently infected cells and in cells undergoing lytic replication and the results showed that KSHV transcripts harbor wide-spread m^6^A modifications in both latent and lytic replication, of which YTHDF2, the ‘reader’ protein, responsible for multiple m^6^A function was found to inhibit lytic replication by enhancing stability of KSHV transcripts (Tan and Gao 2018; Tan *et al.*^2017^; Ye *et al.*^2017^).

### Posttranscriptional Regulation of Latent RNA Transcripts

KSHV major latent locus is positioned at the minus strand of the double-stranded KSHV genome and consists of ORF73 (LANA)/ORF72 (v-Cyclin)/K13 (v-FLIP) and K12 (Kaposin). Posttranscriptional processing of the latent transcripts represents one of the best examples of such regulation during latent phase of KSHV infection. The latent locus expression is initiated from three alternative promoters, LT_c_ and LT_i_ upstream of ORF73 and LT_d_ upstream of ORF72. Both LT_c_ and LT_d_ are constitutive promoters, whereas LT_i_ is an RTA-inducible promoter (Pearce *et al.*^2005^). The primary RNA transcripts initiated from any one of the three latent promoters are tricistronic and polyadenylated either at a poly A site at nt 122,069 downstream of K13 or at nt 117430 downstream of K12 (Majerciak *et al.*^2013^). In addition to be regulated by alternative polyadenylation, the LT_c_ trisitronic transcript also contains two introns with the intron 1 bearing two alternative 3′ splice sites for alternative RNA splicing to efficiently and selectively express the ORF72 and K13 ORF. The LT_i_ tricistronic transcript has no intron and may be responsible for LANA expression in the lytic phase. The LT_d_ tricistronic transcript has only one intron and encodes ORF72 and K13 if the intron escapes from RNA splicing, and otherwise, encodes K12 and KSHV viral miRNAs (Ajiro and Zheng 2014). Consequently, these alternative promoter usage, alternative RNA splicing and alternative polyadenylation lead to produce an array of latent mRNA transcripts with different coding potentials for expression of individual ORFs. Although almost all latent mRNA transcripts use cap-dependent translation, K13 may use an internal ribosome–binding site (IRES) for its translation (Bieleski and Talbot 2001). Because splicing of KSHV latent transcripts is required for the expression of KSHV-encoded miRNAs from the intron region, the excised intron spanning nt123595-118799 region must be processed by cellular miRNA processors to produce pre-miRNAs in the nucleus and mature viral miRNA in the cytoplasm (Cai and Cullen 2006; Pearce *et al.*^2005^). Unfortunately, the regulation of posttranscriptional processing of KSHV latent RNA transcripts remains poorly understood to date. One assumption is that there is no viral protein being involved in the posttranscriptional regulation of the latent transcripts.

### Posttranscriptional Regulation of Lytic Gene Transcripts

KSHV genome encodes up to 80 lytic genes for its productive infection, of which more than one-third are split genes with introns and ~65 genes share a single or alternative pA sites with other genes for their RNA polyadenylation (Majerciak *et al.*^2013^). Thus, expression of these lytic genes is regulated by RNA splicing and polyadenylation in addition to transcription regulation. One of the well-studied lytic gene loci is the gene cluster region of RTA (ORF50), K8 and K8.1. All three genes are collineated side-by-side in the plus strand of the double-stranded viral genome and have their own promoters and gene-body introns, but share a single pA site downstream of K8.1. The expression of these three genes are in a cascaded order from immediately early (RTA), early (K8) to late (K8.1) stage of productive infection, but all utilize the same pA site for RNA polyadenylation. Thus, the transcripts from this region are polycistronic for RTA, bicistronic for K8 and monocistronic for K8.1 and subject to multiple levels of posttranscriptional regulation (Tang and Zheng 2002; Zheng 2003).

During lytic KSHV infection, viral genes are efficiently expressed to the levels required for virus replication and production. As described above, KSHV encodes RTA protein as a powerful transactivator to initiate transcription of most, if not all, viral lytic genes by binding to the promoter of individual genes. More importantly, KSHV encodes ORF57 (MTA, mRNA transcripts accumulation) as a powerful posttranscriptional regulator (Kieshner *et al.*^2000^; Majerciak and Zheng 2009) to secure that viral RNA transcripts could be processed properly and become stable, exportable from the nucleus to the cytoplasm and translatable in the cytoplasm. Thus, both RTA and ORF57 are equally essential for the robust expression of KSHV genes. In the absence of ORF57, most viral transcripts derived from RTA-mediated transcription are unstable and in short half-life because of lacking proper RNA processing (Han and Swaminathan 2006; Majerciak *et al.*^2007^, ^2015^).

### ORF57 Expression and Protein Structure

**Fig. 4:**
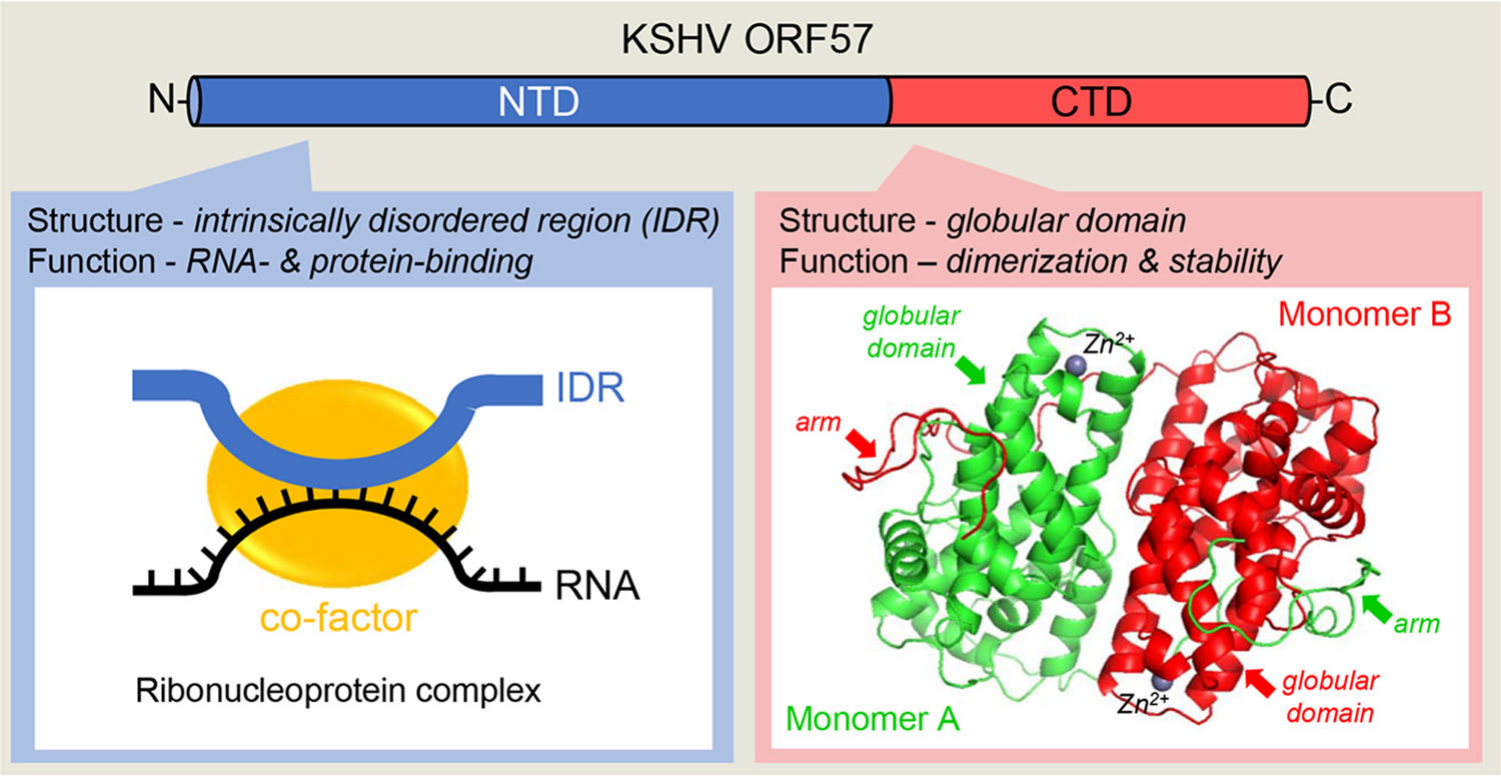
Structural composition of ORF57 protein. KSHV ORF57 contains two structurally and functionally distinct domains. Majority of the N-terminal domain (NTD, in blue) consists of a flexible, intrinsically disordered region (IDR) that binds both target RNA (black line) and cellular cofactors (yellow circle) to form specific ribonucleoprotein complexes. The highly structured C-terminal domain (CTD, in red) contains an alpha–helix-rich globular domain and forms a homodimer stabilized by intermolecular interactions between two monomers (red and green) and Zinc cations (Zn^2+^) binding and then by holding each other with an arm from the NTD of other monomer (Yuan *et al.*^2018^).

**Fig. 5:**
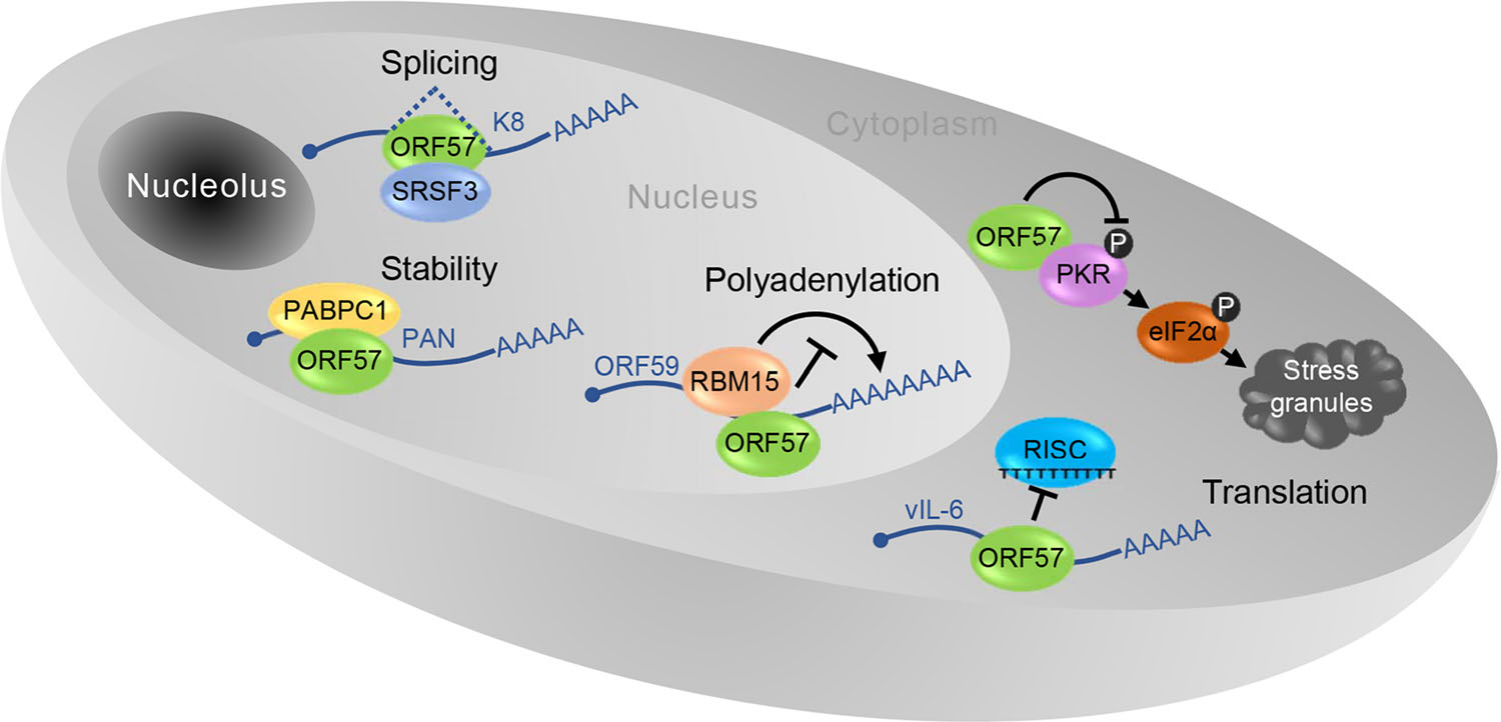
A pleiotropic role of ORF57 in KSHV posttranscriptional regulation. In the nucleus ORF57 prevents hyper-polyadenylation of ORF59 through interaction with RBM15, increases stability of nuclear non-coding PAN RNA via interaction with PABPC1 and stimulates splicing of K8 pre-mRNA by interacting with SRSF3. In the cytoplasm ORF57 promotes vIL-6 translation by preventing miRNA-containing RISC complex binding to vIL-6 mRNA and blocks the formation of stress granules by binding to PKR to prevent PKR activation and phosphorylation (gray circle with P) of translational activator eIF2α.

Expression of ORF57 is activated in the early stage of KSHV infection by RTA binding to the ORF57 promoter (Duan *et al.*^2001^; Lukac *et al.*^2001^; Majerciak and Zheng 2009). The primary ORF57 transcript contains a small, constitutive intron in its 5′ half and a large, suboptimal intron in its 3′ half for alternative RNA splicing and is polyadenylated at a pA site 92 nts downstream of its coding region. This pA site is also used for expression of ORF56 upstream of ORF57 (Majerciak *et al.*2006b). The full-length ORF57 mRNA is derived from RNA splicing of the constitutive small intron, but retains the suboptimal large intron as the coding region. During KSHV lytic infection, only a small fraction (< 5%) of ORF57 RNA transcripts undergo double RNA splicing to produce a smaller, noncoding RNA isoform (Majerciak and Zheng 2016). ORF57 composes 455 amino acid residues and exhibits limited sequence homology to its homologues in other herpesviruses (Majerciak and Zheng 2009), but not at all to any known host proteins. Recent biochemical and crystallography studies revealed that ORF57 protein consists of two structurally and functionally distinct domains (Majerciak *et al.*^2015^; Yuan *et al.*^2018^) (Fig. 4). The N-terminal domain (NTD) encompasses first 219 amino acid residues and exhibits properties of an intrinsically disordered region (IDR), the naturally unstructured polypeptides with high binding potentials (Mollica *et al.*^2016^). Similar to other IDR-containing proteins, ORF57 NTD represents a multivalent domain allowing ORF57 to interact with target RNA transcripts via protein-RNA interaction, while simultaneously binding to other proteins that act as cofactors (Fig. 4). Formation of these ribonucleoprotein (RNP) complexes is essential for ORF57 posttranscriptional activities. Disruption of the binding sites in the ORF57 NTD abrogates ORF57 activities. The composition of ORF57-containing RNPs varies along with ongoing RNA process status and compartmentation of its RNA targets. ORF57 modulates both intron-containing and intronless RNA maturation and function by interaction with different cofactor (Boyne *et al.*^2008^; Jackson *et al.*^2011^; Majerciak *et al.*^2008^, ^2011^, ^2014^; Malik *et al.*^2004^; Massimelli *et al.*^2011^) (Fig. 5). The remaining residues (aa 220–455) of ORF57 form a structurally well-defined helix-rich (11 α-helixes) globular domain referred as the C-terminal domain (CTD) (Fig. 4). In general, two ORF57 CTDs are aligned antiparallelly as a homodimer by numerous intermolecular interactions between the globular domains of two monomers and further tightly held each other by an arm (aa 184–219) from other monomer’s NTD (Majerciak *et al.*^2015^; Yuan *et al.*^2018^). In addition, the binding of a zinc cation into the zinc-binding pocket in each monomer further stabilizes ORF57 homodimer (Fig. 4) (Yuan *et al*. 2018). Similar dimer structures were recently found in ORF57 homologues (Patel *et al.*^2015^; Tunnicliffe *et al.*^2015^, ^2018^). Functionally, the CTD dimerization is imperative for ORF57 protein stability at least partially by protecting of ORF57 from degradation by host proteasome (Majerciak and Zheng 2015).

### Roles of ORF57 in Posttranscriptional Regulation

**Fig. 6:**
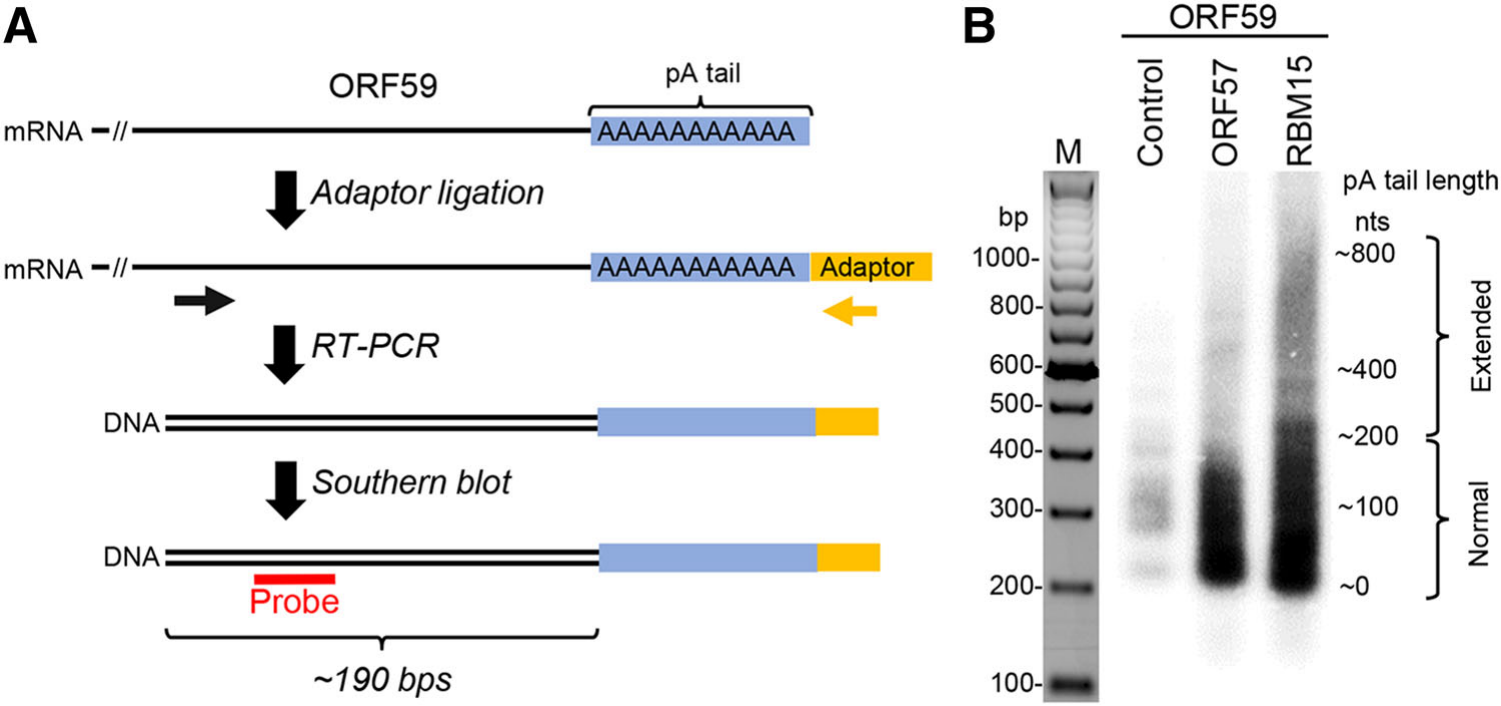
ORF57 prevents RBM15-mediated hyperpolyadenylation of KSHV ORF59 RNA. **A** A workflow of the strategy in determination of RNA poly (**A**) (pA) tail length (blue). First, a chimeric RNA–DNA adaptor (yellow, rUrUrUAACCGCGAATTCCAG/3AmM/-3′) was ligated to the mRNA 3′ end and followed by RT with an adaptor-specific antisene primer (5′-GACTAGCTGGAATTCGCGGTTAAA-3′). The cDNA was amplified by PCR using an ORF59-specific primer (black arrow, 5′-GGATCGTGGGAAGGTGCC-3′) in combination with an adaptor-specific primer (yellow arrow, 5′-GACTAGCTGGAATTCGCGGTTAAA-3′). The ORF59-specific primer is positioned approximately 190 bps upstream of the pA start (black double lines). The obtained PCR products (black double lines) are analyzed by Southern blot using an ORF59-specific probe (red, 5′-AATCAGGGGGTTAAATGTGGT-3′). **B** Length of the ORF59 RNA pA tail in HEK293T cells transfected with a vector expressing ORF59-FLAG fusion protein (pVM18) in the absence (control) or presence of ORF57 or RBM15 was determined by Southern blot as described in (**A**). Total RNA isolated from HEK293T cells was used in this study. The RT-PCR products of ORF59 RNA without a pA tail was about 190 bps in size and the varied sizes or smear signals of the ORF59 cDNA indicate heterogenic length of the pA tails.

Although ORF57 is neither a transcription factor (Massimelli *et al.*^2011^), nor a *bona fide* RNA export factor (Pilkington *et al.*^2012^), its posttranscriptional activities are pleiotropic in nature (Majerciak and Zheng 2015) (Fig. 5). ORF57 promotes the expression of KSHV intronless mRNAs and therefore is historically referred as MTA. ORF57 interacts with cellular export factor Aly/REF and had been thought to act as a viral export factor for increased intronless RNA accumulation (Boyne *et al.*^2008^; Jackson *et al.*^2011^; Malik *et al.*^2004^). Subsequent studies showed that ORF57 interaction with Aly/REF is not a prerequisite for intronless RNA accumulation (Majerciak *et al.*2006a; Nekorchuk *et al.*^2007^). Instead, ORF57 accumulates the viral RNA transcripts by increasing RNA stability primarily through its two major functions: (1) ORF57 binds to the MTA-responsive element (MRE) in its target RNA, such as viral lncRNA PAN (polyadenylated nuclear RNA) and ORF59 (viral DNA polymerase processivity factor) RNA (Fig. 5), to prevent RNA decay (Majerciak *et al.*2006a; Massimelli *et al.*^2011^; ^2015^); (2) ORF57 interacts with cellular RNA-binding proteins RBM15 and OTT3 to prevent RBM15-mediated hyperpolyadenylation of ORF59 (Majerciak *et al.*^2011^) (Fig. 6). In addition, ORF57 also stabilizes the RNA transcripts of KSHV ORF47 (glycoprotein M) and ORF56 (viral primase) (Majerciak *et al.*2006b; Massimelli *et al.*^2013^; Pilkington *et al.*^2012^).

KSHV ORF57 functions as a viral splicing factor and promotes RNA splicing of viral pre-mRNA transcripts in viral lytic infection (Majerciak *et al.*^2008^) (Fig. 5). As described above, almost one-third of ~90 viral genes are split genes with one or more introns, of which RNA splicing is a vital part of their posttranscriptional processing (Arias *et al.*^2014^; Sharp *et al.*^2002^; Sturzl *et al.*^1999^; Tang and Zheng 2002; Yamanegi *et al.*^2005^; Zheng 2003). In this regard, RTA, K8 and K8.1 are transcribed, respectively, as a tricistronic RTA RNA with 4 introns, bicistronic K8 RNA with 3 introns and monocistronic K8.1 RNA with one intron (Tang and Zheng 2002). All of these three gene transcripts are polyadenylated by using a common pA site downstream of the K8.1 ORF and thus, both K8 and K8.1 coding regions are the 3′ UTR region of RTA. ORF57 binds the K8 intron 2 (RTA intron 3), host splicing factors and nuclear small RNAs to promote splicing of RTA and K8 pre-mRNA transcripts (Majerciak *et al.*^2008^, ^2014^). This stimulatory effect of ORF57 on splicing of this intron depends on its interaction with the cellular splicing factor SRSF3 (or SRp20) which binds to this intron and blocks it from splicing (Majerciak *et al.*^2014^) (Fig. 5).

KSHV ORF57 interferes with host miRNA machinery and regulates RNA stability and translation (Fig. 5). This function of ORF57 is two-fold: binding to miRNA seed matches (binding sites) in the target RNAs and interacting with Ago2 and GW182, two major components of RISC (RNA-induced silencing complex). ORF57 binds to a miR-1293 binding site in the ORF region of viral IL-6 RNA and a miR-608 binding site in the ORF region of human IL-6 RNA to prevent miRNA-mediated inhibition on IL-6 translation (Kang JG *et al*. 2011a, b). ORF57 also binds Ago2 and GW182 to prevent Ago2-GW182 interaction and inhibits the formation of RNA processing bodies (P-bodies) (Sharma N *et al*. manuscript in submission). Another function of ORF57 in promotion of viral protein translation is to suppress stress granule formation during lytic infection by interacting with a double-strand RNA-binding protein kinase R (PKR) and blocking PKR activation and phosphorylation of eIF2α, an eukaryotic translation initiation factor (Sharma *et al.*^2017^).

## KSHV Life Cycle and Oncogenesis

The close association of KSHV with some human malignancies (KS, PEL and MCD) has caused virologists to consider KSHV as a human oncovirus. The highlights of KSHV infection are a higher cell growth rate with an extended lifespan, altered cell morphology, deregulated angiogenesis, elevated inflammation and immune evasion to support tumor growth (Dittmer and Damania 2016; Fatahzadeh 2012; Kaplan 2013; Radu and Pantanowitz 2013). The lack of an *in vitro* KSHV cellular transformation model and KS cell lines has hindered research on the mechanisms of KSHV-induced cellular transformation and oncogenesis. Recent progress has been made in KSHV immortalization and transformation of primary rat embryonic metanephric mesenchymal precursor (MM) cells and demonstrated that KSHV-transformed MM cells (KMM) efficiently induce tumors with pathological features similar to those of KS patients (Jones *et al.*^2014^; Moody *et al.*^2013^). Humanized BLT (bone marrow, liver, and thymus) mice infected by inoculation with KSHV.219 virus via the oral and vaginal routes could be a useful model for understanding the pathogenesis and transmission of KSHV (Wang *et al.*^2014^). Further, more evidences have been acquired that KSHV can also infect and induce pathological phenotypes in human mesenchymal stem cells, which make the cells as potential origin of KS tumor cells apart from the previous accepted endothelial cell lineage (Lee *et al.*2016c; Li *et al.*^2018^).

**Fig. 7:**
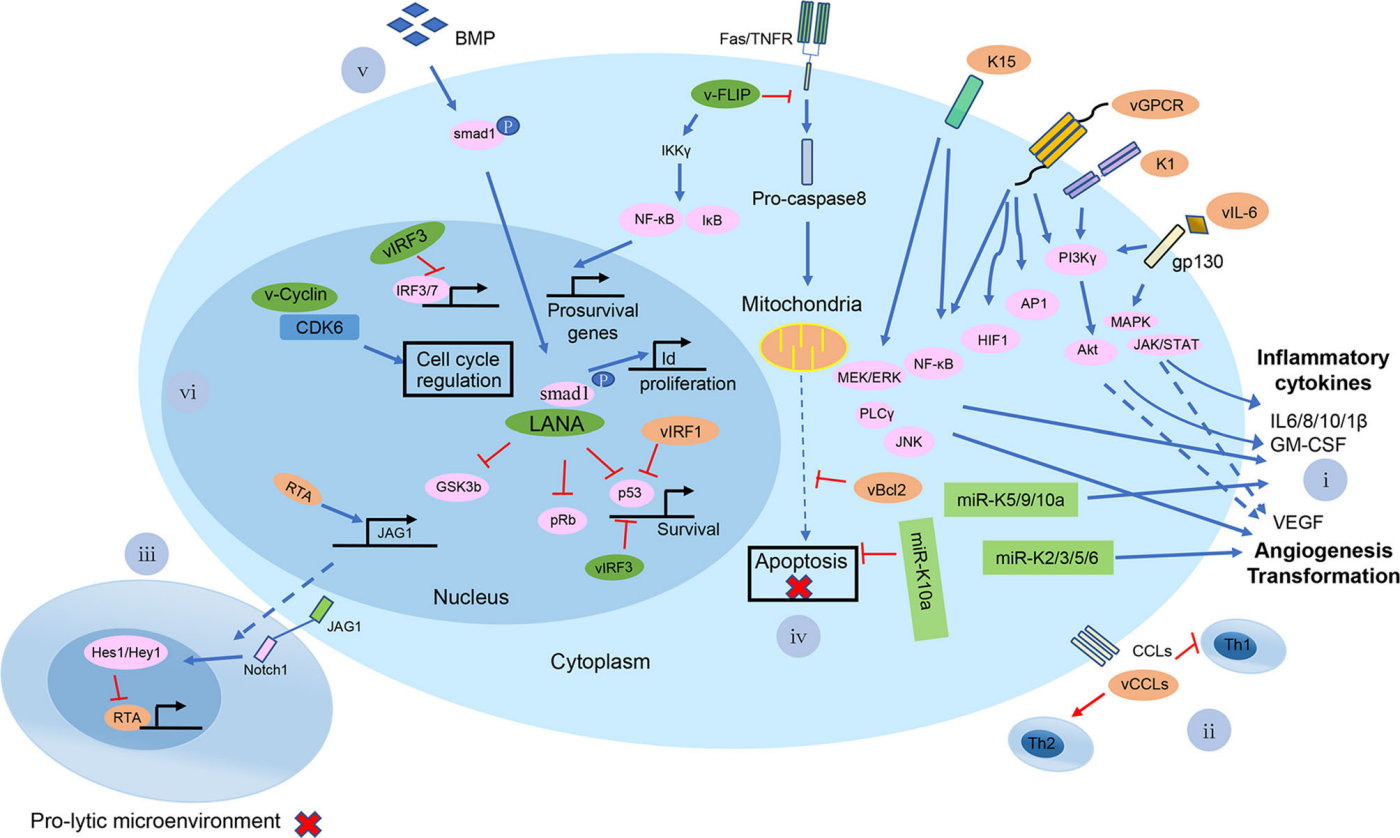
A brief summary of the possible mechanisms of KSHV-mediated pathogenesis at the cellular level. Shown in the diagram are viral lytic proteins in orange and viral latent proteins in green. (i) KSHV vGPCR, K1, K15, and vIL-6 proteins activate the PI3 Kγ/AKT, MAPK, JAK/STAT, NF-κB, MEK/ERK/JNK signaling pathways for infected cells to secrete a number of cytokines and chemokines, such as IL-6/8/10/1β, GM-CSF and VEGF; KSHV miR-K5/9/10a also induced the cytokines secretion. miR-K2/3/5/6 can induce angiogenesis-related factors, like VEGFA; (ii) KSHV encodes three chemokine homologs: viral CC-chemokine ligand-1-3 (vCCL1-K6, vCCL2-K4 and vCCL3-K4.1) to downregulate cellular immune response; (iii) KSHV RTA up-regulates the Notch ligand JAG1 by interacting with LEF1 and triggers Notch activation in neighboring cells. The activated Notch inhibits KSHV reactivation in those neighboring cells. It provides an insight into the mechanism by which a minority of viruses undergoes reactivation, while the majority maintains a persistent latent infection in KS tissues; (iv) v-FLIP in the infected cells inhibits the activation of pro-caspase-8 and has the ability to induce the expression of anti-apoptotic proteins via activation of NF-κB to upregulate pro-survival genes expression. vBcl-2, a homolog of cellular Bcl-2, inhibits apoptosis via tightly binding proapoptotic peptides. KSHV miR-K10a blocks TWEAK-induced apoptosis; (v) LANA affects the BMP signaling pathway and converts it to an oncogenic BMP-Smad1-Id pathway, which might contribute to the pathogenesis of KSHV-induced malignancies; (vi) KSHV LANA binds to and blocks p53-transcriptional activity to inhibit p53-induced cell death. LANA also binds and inactivates pRb, thereby removing the inhibition of pRb-induced cell cycle arrest. LANA binds to GSK-3β and thus accumulates unphosphorylated β-catenin, which in turn affects the cell cycle. v-Cyclin regulates the cell cycle and cell proliferation by constitutive activation of cellular cyclin-dependent kinase 6. vIRF-3 acts as a transcriptional activator on genes controlled by cellular IRF-3 and IRF-7. Further, vIRF-3 negatively regulates p53 protein stability, thereby inhibits p53-mediated activation of p21 gene transcription.

The evidence that most KS spindle cells are latently infected with KSHV suggests that latency may contribute to KSHV-induced malignancies and pathogenesis. Moreover, a small group of cells with latent KSHV infection has been found undergoing lytic replication and producing virus, which suggests that lytic replication may also be important for tumor development (Fig. 7). Thus, most likely, both latent and lytic phases of the virus life cycle are involved in tumor initiation and progression, but their underlying mechanisms could be very different.

KSHV infection can elicit many cellular pathways to facilitate cell survival and proliferation and thus promotes tumor development. LANA, the most important latent protein that is essential for virus maintenance, has been shown to interact with p53 and Rb, the common tumor suppressors (Rivas *et al.*^2001^; Shin *et al.*^2006^). This interaction inhibits cell death and the cell cycle checkpoint, thus contributing to KSHV-induced oncogenesis. KSHV v-Cyclin, another latent protein, has been demonstrated to accelerate cell proliferation by binding to phosphorylated CDK6 (Sarek *et al.*^2006^).

To better maintain tumor growth, KSHV has also evolved strategies to avoid the apoptosis of dysregulated cellular pathways. Many studies have found that v-FLIP, a latent protein, targets the classical NF-κB pathway to promote tumor cell survival. Moreover, NF-κB activation not only leads to cellular transformation, but also increases the incidence of lymphoma in transgenic v-FLIP mice (Guasparri *et al.*^2004^). The inhibition of the NF-κB pathway using specific inhibitors was found to suppress tumor growth in mouse models and in tissue culture *in vitro* (Grossmann *et al.*^2006^; Matta *et al.*^2003^; Ye *et al.*^2008^).

KS is a highly angiogenic neoplasm characterized by heavily and irregularly shaped blood vessels, indicating that during KSHV infection, many angiogenic and lymphangiogenic factors are produced at a relatively high level (Boshoff 2002; Orenstein 2008). Unlike the general action of the host, pathological angiogenesis is correlated with tumor growth and metastasis (O’Byrne *et al.*^2000^; Sakakibara and Tosato 2009), and while the mechanisms of angiogenesis in KS tumor development are not yet clearly understood, KSHV-induced angiogenic factors and inflammatory cytokines appear to be essential for driving KS tumor development (Dimaio and Lagunoff 2012; Purushothaman *et al.*^2016^). The angiogenic cytokines IL-6, IL-8, basic fibroblast growth factor (b-FGF), ephrin B2, cyclooxygenase-2 (Cox-2), heme oxygenase-1(HO-1), Ang2 and MMPs could be induced after KSHV infection (Botto *et al.*^2015^; Cianfrocca *et al.*^2002^; Dai *et al.*^2016^; Haddad *et al.*^2008^; McAllister *et al.*^2004^; Meade-Tollin *et al.*^1999^; Paul *et al.*^2013^; Sadagopan *et al.*^2009^; Sharma-Walia *et al.*^2010^; Ye *et al.*^2013^). Additionally, a number of KSHV-encoded proteins, such as vIL-6, vCCL-1, vCCL-2 and vGPCR, have been shown to act together with vIRF1, v-FLIP, v-Cyclin to stimulate angiogenesis (Asou *et al.*^1998^; Jones *et al.*^1999^; Wu *et al.*^2014^). Furthermore, KSHV-encoded miRNAs could induce angiogenesis by activating multiple cellular signaling (Li *et al.*2016c, 2017; Ramalingam *et al.*^2015^).

To avoid attack from cellular factors, KSHV exploits immune evasion strategies to promote tumorigenesis (Moore and Chang 2003). KSHV encodes multiple proteins which directly or indirectly inhibit cellular immune-related processes and signaling, such as cytokine secretion, antigen processing and presentation, interferon signaling and complement system (Cai *et al.*^2010^). KSHV encodes viral IL-6, which directly binds to gp130, and activates MAPK serine/threonine kinase pathways (Cousins and Nicholas 2013; Molden *et al.*^1997^). To interfere with the complement system, ORF4, also called complement control protein (KCP), blocks progression of the complement cascade (Okroj *et al.*^2011^; Spiller *et al.*^2003^). KSHV encodes four viral homologs of IRF (vIRF1–4) (Baresova *et al.*^2013^). vIRF1 represses cellular IFN-mediated signal transduction by directly interacting with the IFN-stimulated response DNA element and contributes to cell transformation in nude mice to promote tumorigenesis (Baresova *et al.*^2013^). Another important immune evasion strategy exploited by KSHV is downregulation of cell surface MHC class I molecules, and ORF K3 (MIR1) and ORF K5 (MIR2) modulate MHC I molecules on infected cell surfaces via ubiquitylation by acting as E3 ligases (Brulois *et al.*^2014^; Ishido *et al.*^2000^). KSHV encodes several viral chemokines (vCCL) to evade adaptive immunity (Hu and Usherwood 2014). KSHV modulates Toll-like receptor signaling and PKR pathways to disrupt the innate immunity response to virus infection (Lee *et al.*2016b; Sharma *et al.*^2017^). Recently, several reports showed that KSHV encodes ORF52, an abundant tegument protein, which directly binds to cytosolic DNA sensor cGAS subverting its enzymatic activity, thus enabling KSHV to escape the host immune response (Li *et al.*2016a; Wu *et al.*^2015^) (Fig. 7).

## Perspectives

More than two decades after KSHV discovery, the joint efforts of scientists worldwide have propelled numerous advances in every aspect of KSHV research, but many questions obviously remain. Up to date, multiple KSHV genomes from infected B cells, tumor tissues, purified virions and BAC-derived genomes have been sequenced and ~ 81 ORFs have been roughly annotated to a ~137-kb long unique region (LUR) according to a continuous stretch of > 100 codons that begins with a start codon (usually AUG) and ends at a stop codon (usually UAA, UAG or UGA). Although this ORF annotation was initially useful for our quick start to understand the functions of individual viral proteins by using an overexpression system, the caveat is that KSHV bears a complex genome having many split genes and the current ORF annotation completely ignored the discontinuous feature of these split genes in the KSHV genome. Thus, the published studies based on the initial annotated ORFs on split genes were not reliable and misleading the field. Moreover, many annotated genes are clustered in a locus to share a common pA site downstream for their expression (Majerciak *et al.*^2013^) and the annotated ORF(s) downstream in the gene cluster locus could be the 3′ untranslated region (UTR) of other gene (s) upstream. A special precaution has to pay on these gene structures and expression, especially when siRNA or gene knockout approaches are chosen. Repeatedly, we saw many laboratories publishing their studies by using siRNA or gene knockout technologies to target their favorable gene which is also the 3′ UTR region (s) of other gene (s) to draw their “big” conclusion of the targeted gene functions.

Development of the first B cell lines from PEL patients allowed analysis of the viral genome and different aspects of the viral life cycle (Renne *et al.*1996b). Using purified and concentrated KSHV particles, primary endothelial cells had been found to be infected and transformed at the presence of vascular endothelial growth factor A (VEGF) (Flore *et al.*^1998^). Limited by the PEL cells origin, the impact of viral replication on endothelial function or proliferation hadn’t been well studied. Telomerase-immortalized microvascular endothelial cells (TIME) with KSHV virions had been found to support efficient latent infection and reagents-induced lytic growth and infectious progeny generation (Lagunoff *et al.*^2002^). To further elucidate details of the virus life cycle, especially the lytic phase, under relatively physiological conditions, a tetracycline-inducible expression system (TREx BCBL1-Rta) was developed in KSHV-infected PEL cells (Nakamura *et al.*^2003^). Another cell line, iSLK.219 cells, was developed to study latent viral gene expression with tightly controlled and inducible reactivation machinery (Myoung and Ganem 2011a). Moreover, manipulation of the viral genome using the BAC-mediated recombinant system has become more efficient and maneuverable, thus making in-depth functional studies on viral genes and virus-host interactions possible (Brulois *et al.*^2012^; Zhou *et al.*^2002^). Results obtained using KSHV-transformed MM cells suggest that these cells are useful for studying mechanisms of KSHV-induced growth deregulation and oncogenesis (Jones *et al.*^2012^; Moody *et al.*^2013^). To study the viral pathogenesis *in vivo*, many methods have been utilized to develop animal models ranging from severe combined immunodeficient (SCID) mice to *Rhesus macaques*, marmosets, and mice (Chang *et al.*^2009^; Picchio *et al.*^1997^; Renne *et al.*^2004^). However, due to the host specificity of the virus, attempts to develop a better system are continuing.

Although these powerful tools have been developed and the regulatory mechanisms underlying the KSHV life cycle and related pathogenesis have become increasingly clear, many questions need to be addressed. For example, why is latency the default viral gene expression pattern after KSHV primary infection? What detailed and precise regulatory events occurring during the early stage of infection contribute to establishing the so-called default latency? What is the basis for a relatively active viral gene expression pattern instead of typical latency in certain cell types? What are the intrinsic physiological stimuli triggering lytic reactivation from latency? We believe that answering these questions will help better understand regulation of viral life cycles and related pathogenesis, and eventually benefit the development of new therapeutic strategies for KSHV-associated diseases.
